# Suicidal ideation, attempt and associated factors among people living with cancer in Ethiopia: a cross-sectional study

**DOI:** 10.1186/s12991-022-00407-0

**Published:** 2022-07-26

**Authors:** Alemayehu Molla, Mekidem Aderaw, Haregewoin Mulat, Biruk Fanta, Goshu Nenko, Aynishet Adane

**Affiliations:** 1 College of Medicine and Health Science, Comprehensive Specialized Hospital, University of Gondar, Gondar, Ethiopia; 2 Department of Psychiatry, School of Medicine, College of Medicine and Health Science, University of Gondar, Gondar, Ethiopia; 3 Department of Internal Medicine, School of Medicine, College of Medicine and Health Science, University of Gondar, Gondar, Ethiopia; 4 Department of Psychiatry, College of Health and Medical Science, Dilla University, Dilla, Ethiopia

**Keywords:** Suicidal ideation, Suicidal attempt, Cancer, Ethiopia

## Abstract

**Background:**

Suicide and cancer are serious public health problems worldwide, and people living with cancer are at high risk of having suicidal behaviors, such as ideation, plan and attempt. Patients with cancer had high possibilities of having suicidal ideation and attempt which lead to poor adherence of medication, worsening of their medical illness, and end the life. Even though people are affected by cancer in Ethiopia, there are limited studies regarding suicidal problem among patients with cancer. Therefore, this study was aimed to assess the magnitude and associated factors of suicidal ideation and attempt among people living with cancer in Ethiopia.

**Methods:**

Institutional-based cross-sectional study was conducted among total of 416 participants. Outcome variables were assessed using suicidality module of World health organization (WHO) composite international diagnostic interview (CIDI). Data were analyzed using SPSS-20 and bivariate and multivariate logistic regressions were conducted and variables with *P* value less than 0.05 were considered as statistically significant with corresponding 95% CI.

**Results:**

The overall magnitude of suicidal ideation and attempt were 16.6% and 5.5%, respectively. Being divorced [(AOR = 2.97, (95% CI 1.22, 7.22)], having depression [(AOR = 2.67, (95%CI 1.34, 5.32)], the first 18 months, since diagnosed cancer [(AOR = 2.57, (95%CI 1.15, 5.75)], severe pain [(AOR = 3.27, (95%CI 1.18, 9.04)] and stage IV cancer [(AOR = 3.35, (95%CI 1.26, 9.04)] were significantly associated with suicidal Ideation. Whereas, female sex [(AOR = 5.32, (95%CI 1.39, 20.25)], having depression [(AOR = 4.8, (95%CI 1.23, 18)] and advanced stage of cancer [(AOR = 6.76, (95%CL 1.2, 37)] were significantly associated with suicidal attempt.

**Conclusions:**

The magnitude of Suicidal ideation and attempt in this study were high. Health care providers working in cancer treatment unit should give more attention to patients with high suicidal risk factors. Consultation services should be strengthened with psychiatric professionals in oncology treatment clinic.

## Introduction

Suicide is a fatal act of ending one’s own life with some evidence that the person planned to die [1]. Suicidal ideation is defined as thoughts, fantasies, and wishes to engage in any suicidal related behaviors [2, 3]. Suicidal attempt is self-injurious behavior with a non-lethal result, but with some evidence of intention to die [4]. Both suicidal ideation and attempt are major risk factors for completed suicide [5]. Globally more than 25% of individuals experience suicidal ideation, and 10–20 million attempt suicide [6]. World Health Organization (WHO) report showed one individual death in every 20 s by suicide [7, 8]. Suicide is also 10th leading cause of death worldwide and the 2nd causes of death for those 15–29 years [5, 9].

Cancer is the leading cause of morbidity and 3rd leading cause of mortality with 18.1 million new cancer cases and 9.6 million deaths worldwide [10, 11]. The burden of cancer is high in low- and middle-income countries due to change in lifestyle, such as smoking [12], work related risk factors [13], and dietary risks [12]. Ethiopia is also home to a growing population of more than 110 million people with an estimated parallel rise in cancer burden [14]. Cancer has widespread consequences on an individual’s physical, emotional, and spiritual well-being [15, 16]. Intense feelings such as sadness, depression, fear of disability, pain and even suicidal feeling may occur at a time of disclosure of diagnosis and treatment options [16]. Suicidal thought in patients with cancer can be caused due to psychological reaction during diagnosed cancer, long duration of treatment, repeated hospitalizations, diminished quality of life, limited physical activities, and immunological disturbances [17, 18]. Treatment-related factors such as side effects of chemotherapeutic agents and radiation can also contribute to the development of depression and suicidal thoughts [19].

Several studies identified that the magnitude of suicidal ideation and attempt among patients with cancer is 2–5 times higher than general population [9, 19, 20]. The magnitude of suicidal ideation among people living with cancer ranges 12.85% in USA [21] to 71.4% in South Africa [22]. Whereas, suicidal attempt ranges 0.4 in Canada [23] to 14.6% in China [24].

Studies conducted in different countries showed that; male gender, older adults, change in marital status, family history of psychiatry disorder, early life adversity, chronic medical illnesses, chronic obstructive pulmonary disease, stroke and asthma were statistically associated with suicidal behaviors among patients with cancer [25–27]. The presence of suicidal ideation and attempt in people living with cancer leads to poor compliance with the treatment, worsening of the condition, reduce chances of survival, increase mortality from the diseases, and has a negative impact on the quality of life lead to unsuspected end of their life [9].

In Ethiopia, more than 50% of cancer patient had depression, but only 6.4% are identified and received treatment due to limitation technology, lack of specialized professionals, and patient related factors [28–30]. Studies conducted in Ethiopia indicated high magnitude of Suicidal behavior among patients with severe mental illness and other chronic conditions, such as human immune virus (HIV), Epilepsy, Diabetes, hypertension (HTN) and Tuberculosis (TB), but little attention is given to identify suicide among cancer patients even if the burden of cancer is high from time to time. Therefore, the current study was conducted to assess the prevalence and associated factors of suicidal ideation and attempt among people living with cancer in Ethiopia.

## Methodology

### Study design and setting

Institution-based cross-sectional study was conducted from April 28 to June 5, 2020 at the Comprehensive Specialized Hospitals of University of Gondar and Felege Hiwot Referral Hospital. University of Gondar Specialized Hospital is found 723 km far from Addis Ababa, Capital city of Ethiopia, and providing clinical service for approximately 7 million peoples around the catchment areas [31]. Felege Hiwot referral Hospital is found at Bahir Dar town 558 km far from Addis Ababa in the Northern part of Ethiopia. This Hospital is also Referral center for district hospitals and gives clinical services for more than 5.5 million people.

### Study participants

All people Diagnosed cancer and visiting the outpatient department at University of Gondar and Felege Hiwot referral hospital during data collection period were the study participants. Participants were interviewed after obtaining ethical clearances from University of Gondar. Written consent form was also obtained from participants, and all information obtained was kept confidential during all stages of the study. The collected data were used only for the purpose of the study.

### Inclusion and exclusion criteria

All people Diagnosed cancer visiting the outpatient department at University of Gondar and Felege Hiwot referral hospital with age range of 15 years and above were included in the study, whereas critically ill patients who were unable to communicate due to the severity of the illness during the data collection period were excluded.

### Sampling

The sample was calculated using single population proportion formula $ni=\frac{({\frac{Za}{2})}^{2} \times p\left(1-p\right)}{{d}^{2}}$ with 5% margin of error, 95% confidence level, 10% non-response rate and considering proportion of suicide as 50% among cancer patients, the final sample size was 423. A systematic random sampling technique was used to select samples after proportionally allocating for two hospitals.

### Instruments

Socio-demographic characteristics of respondents were collected by structured socio-demographic questionnaires, clinical factors were collected by semi-structured questionnaires and reviewing the patient’s medical charts.

Suicidal ideation and attempt were assessed using the suicidality module of the World Mental Health (WMH) survey initiative version of the World Health Organization (WHO) Composite International Diagnostic Interview (CIDI) [32]. It contains a module that assesses the lifetime, last 12 months, and 1 month occurrence of suicidal behavior (suicidal ideation, attempt, and plan). It also assesses the methods used to attempt suicide, the patient’s reason to attempt suicide, and their response after attempt. Tool was validated and used for both clinical and community settings in Ethiopia.

Social support was assessed by 3 item Oslo social support scale, it is commonly used to assess social support and it has been used in several studies, the sum score scale is ranging from 3 to 14, which has 3 categories: poor social support 3–8, moderate support 9–11and strong social support 12–14 [33]. Depression and anxiety were assessed using the Hospital Anxiety and Depression scale (HADS) [34], it is a tool developed to evaluate depression and anxiety symptoms in medical patients. It has a 14 item score composed of two subscales for assessing anxiety and depression. The questions relating to anxiety and depression are marked ‘A’ and ‘D’, respectively. Patients rate each item from 0 to 3 which ranges from 0 to 21 for each subscale. Clinically significant anxiety and depression were defined as a cutoff 8 [34].

### Data collection

Data were collected by both faces to face interviews and reviewing the patient’s medical charts using Amharic version of pre-tested questionnaire. Training was provided to data collectors and supervisors for 2 days on sampling procedures, ethical issues and confidentiality. Daily supervision and reviewing of the collected data were done by the investigators.

### Data processing and analysis

The coded and checked data were entered into the computer using Epi data version 6.4 and imported to statistical package for social science (SPSS) version 20. Descriptive statistics such as frequency, percentage, and mean were computed and presented using tables, and texts to show a picture of the data. Bivariate and multivariate logistic regression analyses were conducted to determine the presence of a statistically significant association between explanatory variables and outcome variables. Variables with *P* value less than 0.05 were considered statistically significant and the strength of the association was presented by odds ratio with 95%C.I.

## Result

### Socio-demographic characteristics of participants

A total of 416 participants were included with a response rate of 98.5%. The median age of participants was 50 years with Inter Quartile Range of (IQR 37–60 years). More than half of the respondents were females 228 (54.8%).The larger proportion of participants, 372 (89.4%) were Amhara by ethnicity and more than two-thirds of respondents 280 (67.3%) were Orthodox religious followers. Approximately two third, 280 (68.8%) of the respondents were married. With respect to the educational status of respondents, about 162 (38.9%) of them had not attended formal education and nearly one-fourth of respondents 107 (25.7%) were employed. The living arrangement of respondents indicated that three-fourth 312 (75%) of them were living with their own families (Table1).

**Table 1:** Socio-demographic characteristics of people living with cancer in, Ethiopia 2020 (*n* = 416)

Variables	Category	Frequency	Percentage
Age	16–30	67	16.1
	31–64	264	63.5
	≥65	85	20.4
Sex	Male	188	45.2
	Female	228	54.8
Religion	Orthodox	280	67.3
	Muslim	64	15.4
	Protestant	54	13.0
	Catholic	10	2.4
	Other*	8	1.9
Marital status	Single	67	16.1
	Married	286	68.8
	Divorced	45	10.8
	Widowed	18	4.3
Ethnicity	Amhara	372	89.4
	Oromo	11	2.6
	Tigray	21	5.0
	Other**	12	2.9
Educational status	No formal education	162	38.9
	1–8 Grade	91	21.9
	9–10 Grade	43	10.3
	11–12 Grade	24	5.8
	Diploma	77	18.5
	Degree and above	19	4.6
Occupation	Employed	107	25.7
	Merchant	82	19.7
	Farmer	98	23.6
	Student	34	8.2
	Daily worker	11	2.6
	Housewife	67	16.1
	Retired	17	4.1
Living arrangement	Living with families	312	76.9
	Living alone	58	12.0
	Living with friends	28	6.7
	Living with others	18	4.3

Other* Jewish, 7 day Adventist, Other** Benishangul Gumuz, Afare, Agew

### Clinical and psychosocial characteristics of the respondents

Regarding clinical factors, the most common cancer types were breast 105 (25.2%) and gynecological 68 (16.3%), respectively. More than half, 244 (58.7%) of participants were below 18 months since diagnosed cancer. Relatively large numbers of participants were with the diagnosis of stage III cancer 115 (27.6%). About three-fourth 321 (77%) of the respondents had moderate-to-severe pain. The nearly half 225 (54.1%) of participants hade moderate social support, two-in-five, 167 (41.1%), and one-third of the total participants 122 (29.3%) had co-morbid depression and anxiety, respectively (Table2).

**Table 2:** Clinical characteristics of people living with cancer in Ethiopia, 2020 (*n* = 416)

Variable	Category	Frequency	Percentage
Anatomical site of cancer	Breast	105	25.2
	Genitourinary	15	3.6
	GI	52	12.5
	Gynecological	68	16.3
	Hematological	37	8.9
	HNC	62	14.9
	Lung	29	7.0
	Other*	48	11.5
Time since diagnosis	≤18 months	244	58.7
	> 18 months	172	41.3
Stage of cancer	Stage I	98	23.8
	Stage II	105	25.2
	Stage III	115	27.4
	Stage IV	98	23.6
Severity of pain	No pain	10	2.4
	Mild pain	85	20.4
	Moderate pain	220	52.9
	Severe pain	101	24.3
Type of treatment	not start treatment yet	28	6.7
	Chemotherapy	237	57.0
	Surgery	105	25.2
	Palliative care	5	1.2
	Combination therapy	27	6.5
	Other**	14	3.4
Chronic medical illness	Yes	73	17.5
	No	343	82.5
Taking other medication	Yes	69	16.6
	No	347	83.4
Depression	Yes	167	41.1
	No	249	58.9
Anxiety	Yes	122	29.3
	No	294	70.7
Known mental illness	Yes	6	1.4
	No	410	98.6
Family history of mental illness	Yes	34	8.2
	No	382	91.8
Family history of suicide	Yes	9	2.2
	No	407	97.8
Social support	Poor social support	86	20.7
	Moderate social support	225	54.1
	Strong social support	105	25.2

Other* Pancreatic cancer, Sarcoma, Liver cancer, Skin cancer, other** hormonal and radiation therapy

### Prevalence of suicidal ideation and attempt

The 12 month prevalence of suicidal ideation and attempt were 69 (16.6%) with (95% CI 13–20) and 23 (5.5%) with (95% CI 3.4–7.9), respectively. With respect to frequency of suicidal attempt, 17 (73.9%), 4 (17.4%), and 2 (8.7%) attempted suicide once, twice and more than two times, respectively. The majority of suicide attempters 16 (69.9%) reported that their suicide attempt was related to the current physical illness and 12 (52.2%) of them made serious attempts. The most common method of attempt was poisoning 14 (60.9%) followed by hanging 6 (26.1%) (Table 3).

**Table 3:** Prevalence of Suicidal ideation and Attempt among people living with Cancer in Ethiopia, 2020 (*n* = 416)

Variable	Category	Frequency	Percentage
Life time suicidal ideation	Yes	124	29.8
	No	292	70.2
One year suicidal ideation	Yes	69	16.6
	No	347	83.4
one month suicidal ideation	Yes	52	12.5
	No	364	87.5
Life time suicidal attempt	Yes	61	14.7
	No	355	85.3
One year suicidal attempt	Yes	23	5.5
	No	393	94.5
One month suicidal attempt	Yes	13	3.1
	No	403	96.9
Life time suicidal plan	Yes	41	9.9
	No	375	90.1
One year suicidal plan	Yes	33	7.9
	no	383	92.1
One month suicidal plan	Yes	3	0.7
	No	413	99.3
Frequency of suicidal attempt	Once	17	73.9
	Two times	4	17.4
	More than two times	2	8.7
Reasons for suicidal attempt	Physical illness	16	69.6
	Family conflict	2	8.7
	Poverty	5	21.7
Method of suicidal attempt	Hanging	6	26.1
	Poisoning	14	60.9
	Using sharp tools	3	13
Reasons that describe patients response	Made serious attempt	12	52.2
	Methods used was not effective	8	34.8
	Attempt was to seek help	3	13

### Associated factors of suicidal ideation and attempt

In multivariate logistic regression; being divorced, having depression, less than 18 months since diagnosis, presence of severe pain and stage IV cancer were found to be significantly associated factors with suicidal ideation at *P* < 0.05.

The odds of having suicidal ideation among divorced patients was 2.97 (AOR = 2.97, 95%CI 1.22, 7.22) times higher compared to married participants. The odds of having suicidal ideation for those under 18 months of their cancer diagnosis was 2.5 (AOR = 2.5, 95%CI 1.15, 5.75) times higher compared to participants diagnosed cancer for above 18 months. The odds of suicidal ideation among patients with stage IV cancer was 3.35 (AOR = 3.35, 95%CI 1.26, 9.04) times higher as compared to stage I.

The suicidal ideation is 3.27 times higher among patients with severe pain (AOR = 3.27, 95%; CI 1.18, 9.04). Patients with depression were 2.67 (AOR = 2.67, 95%CI 1.34, 5.32) times more likely to develop suicidal ideation as compared with their counterparts (Table 4).

**Table 4:** Associated Factors of Suicidal ideation among people living with cancer in Ethiopia 2020 (*n* = 416)

Variable	Category	Suicidal ideation		COR (95% CI)	AOR (95% CI)
		Yes	No		
Sex	Male	22	166	I	I
	Female	47	181	1.95(1.13,3.39)	1.57(0.81,3.03)
Marital status	Single	9	57	0.9(.41,1.95)	1.01(0.4,2.7)
	Married	43	245	I	I
	Divorced	13	31	2.38(1.15,4.9)	2.97(1.22,7.22)*
	Widower	4	14	1.62(0.51,5.18)	0.61(0.14, 2.67)
Time since diagnosis	≤ 18 months	58	186	4.56(2.31,8.99)	2.57(1.15,5.75)**
	> 18 months	11	161	I	I
Stage of cancer	Stage I	8	91	I	I
	Stage II	9	96	1.06(0.39,2.88)	1.17(0.3,3.54)
	Stage III	16	98	1.85(0.75,4.54)	1.08(0.38,3.04)
	Stage IV	36	62	6.60(2.87,15.16)	3.35(1.26,9.04)*
Severity of pain	NO/mild pain	7	88	I	I
	Moderate pain	23	197	1.46(0.67,3.54)	0.71(0.25,1.97)
	Severe pain	39	62	7.90(3.32,18.83)	3.27(1.18,9.04)*
Family history of mental illness	Yes	9	25	1.932(0.85,4.34)	0.84(0.29,2.38)
	No	60	322	I	I
Chronic medical illness	Yes	23	50	2.97(1.65,5.32)	2.05(0.99,4.24)
	No	46	297	I	I
Anxiety	Yes	34	88	2.85(1.68,4.85)	1.71(0.88,3.29)
	No	35	259	I	I
Depression	Yes	52	119	5.86(3.24,10.58)	2.67(1.34,5.32)**
	No	17	228	I	I
Social support	Poor	29	57	4.34(2.07,9.37)	1.96(0.73,5.29)
	Moderate	29	196	1.26(0.6,2.64)	1.34(0.53,3.35)
	Strong	11	94	I	I
Current alcohol use	Yes	30	71	2.99(1.73,5.14)	1.5(0.76,2.97)
	No	39	276	I	I

For suicidal attempt, being female sex, having depression, and stage IV cancer were significantly associated factors at *P* < 0.05. The odds of suicidal attempt among female participants was 5.39 (AOR = 5.3, 95%CL 1.39, 20.2) times higher. Patients with depression were 4.8 (AOR = 4.8, 95%CI 1.23, 18.6) times more likely to attempt suicide compared with their counterparts. The odds of suicidal attempt among patients with stage IV cancer were 6.76 (95% CI 1.23, 20.37) times higher as compared to stage I (Table 5).

**Table 5:** Factors associated with suicidal attempt among patients with cancer in Ethiopia 2020 (*n* = 416)

Variable	Category	Suicidal attempt		COR (95% CI)	AOR (95% CI)
		Yes	No		
Sex	Male	3	185	I	I
	Female	20	208	5.92(1.73,20.27)	5.32(1.39,20.25)*
Severity of pain	No/mild	1	94	I	I
	Moderate	9	211	4(0.5,32.1)	1.58(0.16,14.96)
	Severe	13	88	13(1.77,108)	2.41(0.24,23.54)
Time since diagnosis	≤18 months	20	224	5.03(1.47,17.2)	1.96(0.48,7.91)
	> 18 months	3	169	I	I
Stage of cancer	Stage I	2	97	I	I
	Stage II	2	103	0.94(0.13,6.81)	1.53(0.18,13.06)
	Stage III	3	111	1.31(0.21,8)	1.02(0.14,7.3)
	Stage IV	16	82	9.46(2.11,42)	6.76(1.20,37)*
Depression	Yes	20	151	10.68(3.12,36.58)	4.8(1.23,18.6)*
	No	3	242	I	I
Anxiety	Yes	14	108	4.1(1.72,9.76)	2.19(0.76, 6.25)
	No	9	285	I	I
Social support	Poor	9	77	1.92(0.65,5.65)	0.62(0.16,2.43)
	Moderate	8	217	0.6(0.2,1.8)	0.52(0.14,2.03)
	Strong	6	99	I	I

^*^P < 0.05, **P < 0.01

## Discussion

This study showed that 16.6% (95%CI 13.0–20.6) of patients with cancer had suicidal ideation. This is in line with other studies done in Italy 20% [35], Korea (8.9% [36], Canada 15.5% [23], China 18.1% [37], and USA 17.1% [38]. However, it was lower than studies done in South Africa 71.4% [39], Spain 25.24% [40], Portugal 34.5% [41], South Korea 24.7% [42] and China 46.3% [43]. On the other hand, the finding of the current study was higher than other studies conducted in Spain 11.7% [44], Canada 9.6% [45], USA 12.4% [46] and in white, non-Hispanic population 8.4% [21].

The possible reason for this difference might be due to the variations in study participants, study setting and sample size. A study done in South Africa used patients with only cervical cancer, while studies done in Portugal and South Korea focused exclusively in patients with the advanced stage of disease and specific cancers found in four sites (lung, breast, cervical, and head and neck), but the current study include various cancer types. The studies carried out in Spain recruited patients who were in the advanced stage of diseases and under palliative care, while the current study focused in the outpatient department [40]. The difference in study deign might be also another possible reason for variations, study conducted in Canada was community-based survey, whereas studies in Spain and USA were longitudinal.

The prevalence of suicidal attempt in this study was 5.5% (95% CI 3.4, 7.9) which is in agreement with studies conducted in Colombia and Korea 4.5% [47] and 4.2% [25], respectively. The finding was higher than other studies conducted Sweden 1.07% [48], Canada 0.4% [23] Korea 2.6% [36] and USA 3.98% [21]. However, the magnitude of suicidal attempt in the current study was lower than studies conducted in Turkey 12.74% [49] Korea 12.7% [42] and China 14.6% [43]. The possible reason for this discrepancy could be due to the difference in study design, study setting, and study participants. A study done in Korea used a self-administered questionnaire, while the study in Sweden used retrospectively reviewed inpatient. Another possible reason might be due to the difference in the study population. A study performed in Korea and China recruited only patients with an advanced stage of cancer, while the current study recruited all stages. This may inflate the magnitude of suicidal attempt, since suicidal behavior is more common in the advanced stage than an early stage. The time difference between the current and previous studies may also be attributable for this variation. Because of better communication, advance in early diagnosis and treatment, and increased overall survival might more likely improve the reaction of a cancer diagnosis.

Regarding factors associated with suicidal ideation, divorced cancer patients were more likely to have suicidal ideation compared to married participants. This finding is consistent with a study done in USA [38]. The possible reason could be that divorced patients lost their primary source of emotional, social, and instrumental support which may lead to less comprehensive social network, isolation and depression [38]. The presence of depression was reported to be associated with suicidal ideation in several studies done in Spain [40], Canada [23], China [50] and USA [46]. Similarly, the current study found that depression is significantly associated with suicidal ideation. This finding is expected because of that depression in cancer patients leads to feelings of despair, heightened distress and hopelessness. It also lowers serotonin levels which causes poor impulse control, sleep disturbance and loss the desire to live. These may leads to negative expectations for the future and thoughts of ending one’s own life [51].

The other factor which increases suicidal ideation was less than 18 months since diagnosed cancer. This is consistent with the studies carried out in Norway [9] and China [29].This might be because of that the first 18 months are a highly critical and stressful period when patients encountered anxiety due to painful emotional reactions after disclosure of diagnosed cancer and treatment options. Because of these patients might consider suicide as a way to escape from their suffering. As time goes on patients may undergone psychological copying ability and accept their diagnosis [21, 50]. The statistically significant association between suicidal ideation and the stage of cancer in this study is supported by studies done in Denmark [9] Korea [42] and India [52]. The possible reason for these could be that advanced stage of cancer is more likely to involve many-body structures, organs and systems, more likely to cause cancer complications such as severe pain, depression, cognitive impairment, sleep disturbance and progressive deterioration in the quality of life which may lead a patient to have a high level of psychological distress [50].

The severity of pain was also reported to be associated with suicidal ideation in previous studies done in Korea [53], Canada [23] and China [50] similar to our study. It could be due to the reason that patients with severe pain develop anxiety because of fear of death, fear of recurrence, and uncertainty to future life. This may in turn lead to thoughts of ending one’s own life as a way to escape from suffering. Drugs given to relieve pain (corticosteroids, narcotics and analgesics) produce restlessness, agitation and depression either during high dose or withdrawal which produce high psychological distress [50, 51].

Regarding factors associated with suicidal attempts being female was about 5.3 times more likely to have suicidal attempts compared to males. This is in agreement with the studies done in Spain [40] and Canada [54]. The reason for these could be that females are more vulnerable to psychosocial stressors and more likely to have depression compared to males [40]. However, there are also other studies which show high prevalence of suicidal attempt among men as compared with their counterparts [55–57].The possible reason for this might be due to cultural influences which play an important role in the suicide, in most cultures men are expected to use lethal method and does not seek advice for their suicidal attempt [57]. Other factor that significantly associated with suicidal attempt is depression. This is supported by a study carried out in USA [48]. The possible reason might be that cancer patients with depression are at high risk of emotional and psychological distress, feelings of hopelessness, and loss the desire to live. Patients with the advanced stage of cancer were more likely to attempt suicide as compared to an early stage of cancer. This is line with studies done in USA [48]. These can be explained by the fact that advanced stage of cancer places individuals at high-risk psychological distress [51].

## Limitation of the study

Because of the nature of study design making an inference on the causal relationship between suicidal ideation, attempt, diagnosis of cancer and its identified factors is not possible. Some clinical factors such as hopelessness which could determine suicide are not included in this study, and there might be recall bias for some variables.

## Conclusions

This study demonstrated that the magnitude of suicidal ideation and attempt among people living with cancer were high compared with most other studies. This sounds the need to develop and implement suicidal prevention strategies in oncology treatment unit. Training to identify suicidal risk factors should be given for health professional working in oncology treatment unit, and consultation services should be strengthened with psychiatric professionals in oncology treatment clinic.

## Data Availability

The data included in the manuscript can be accessed from the corresponding author through the email address of “alexmolla09@gmail.com” with rational request.

## Abbreviations

AOR: Adjusted odds ratio
CI: Confidence interval
CIDI: Composite international diagnostic interview
COR: Crude odds ratio
SBQ, USA: United States of America
WHO: World Health Organization
